# Chronic Total Occlusion Revascularization Strategies: A Comparative Study of Percutaneous Coronary Intervention and Coronary Artery Bypass Grafting

**DOI:** 10.31083/RCM27226

**Published:** 2025-06-27

**Authors:** Yanci Liu, Shaoping Wang, Hongyu Peng, Qian Fan, Jinghua Liu

**Affiliations:** ^1^Department of Cardiology, Beijing Anzhen Hospital, Capital Medical University, 100029 Beijing, China

**Keywords:** chronic total occlusion, percutaneous coronary intervention, coronary artery bypass grafting, revascularization, stent

## Abstract

**Objective:**

Currently, there are limited data on the clinical outcomes of percutaneous coronary intervention (PCI) compared to coronary artery bypass grafting (CABG) for the treatment of chronic total occlusion (CTO). We compared the clinical outcomes of patients with CTO lesions treated by PCI versus CABG.

**Methods:**

This study included 2587 patients with coronary artery disease (CAD) with CTO from January 1, 2019 to December 31, 2021. Both short- and long-term clinical outcomes were compared in patients with CTO who received successful revascularization. The primary endpoint, defined as major adverse cardiac and cerebrovascular events (MACCE), was a composite of all-cause mortality, cerebrovascular events, and myocardial infarction. Unplanned revascularization and heart failure hospitalization were defined as secondary endpoints separately. Propensity score matching was applied to balance baseline characteristics between the two groups.

**Results:**

The PCI group had lower MACCE (0.47% vs. 2.11%) within 30 days of the index operation, but the difference did not reach statistical significance (*p* = 0.06). After an average follow-up of 37.2 months, no significant differences were observed between PCI and CABG in all-cause mortality (hazard ratio [HR] = 2.29, 95% CI: 0.79–6.61; *p* = 0.13), MACCE (HR = 2.03, 95% CI: 0.86–4.76; *p* = 0.10), or heart failure hospitalization rate (sub distribution HR [SHR] = 0.98, 95% CI: 0.26–3.74; *p* = 0.98). However, patients who underwent PCI had a higher risk of unplanned revascularization (SHR = 10.32, 95% CI: 2.42–43.95; *p* = 0.002).

**Conclusion:**

In patients with CAD with CTO, PCI was associated with a trend of lower short-term MACCE compared to CABG, but with a higher risk of long-term unplanned revascularization. There were no significant differences in long-term all-cause mortality, MACCE, or heart failure hospitalization rates between PCI and CABG.

## 1. Introduction

Chronic total occlusion (CTO) poses a significant technical challenge in interventional cardiology [1]. It is estimated that 15–25% of coronary angiographies reveal at least one CTO lesion [2].

Revascularization of CTO has multiple advantages. First, improving anginal symptoms and quality of life has been demonstrated by clinical trials [3, 4]. Second, an observational study suggests that successful revascularization improves left ventricular ejection fraction (LVEF) and reduces left ventricular end-systolic volume in selected populations [5]. However, the REVIVED–BCIS2 [6] trial found that percutaneous coronary intervention (PCI) did not improve all-cause mortality or left ventricular systolic function in patients with left ventricular systolic dysfunction. Thus, the revascularization of CTO remains a subject of debate. Third, the presence of a CTO increases the risk of ventricular arrhythmias; thus, revascularization may enhance myocardial electrical stability [7, 8]. Lastly, it has been suggested that revascularization of a CTO could potentially reduce the risk of a “double jeopardy” scenario. This occurs when an acute coronary syndrome arises from the sudden occlusion of a non-CTO coronary artery that supplies collateral flow to the myocardial territory of the CTO. Such an event could result in acute multivessel myocardial infarction and increase the risk of circulatory collapse caused by cardiogenic shock [9, 10, 11].

In clinical practice, however, optimal medical therapy remains the primary treatment for the majority of patients with CTO. Only a minority of patients with CTO are believed to undergo coronary artery bypass grafting (CABG) (22–26%) or PCI (10–22%) [12]. CTO PCI patients typically present with a higher prevalence of comorbidities, more risk factors, and a greater incidence of multivessel disease. Cardiologists exercise particular caution when performing revascularization for CTOs due to the prolonged procedure times, increased risk of complications, and lower success rates compared to non-CTO lesions [3].

Success rates of CTO PCI have significantly improved over the past decade with advancements in technology, the adoption of new equipment, and CTO algorithms for revascularization. An observational study revealed that, after adjusting for clinical factors, patients undergoing CTO PCI exhibited a comparable long-term risk of all-cause mortality to those undergoing non-CTO PCI [5]. Therefore, PCI has become an alternative treatment for CTO.

To date, the optimal revascularization strategy for CTO remains controversial. A recent meta-analysis demonstrated that PCI outperformed CABG in reducing all-cause mortality and cardiac death but was less effective in lowering the rates of myocardial infarction and repeat revascularization [13, 14]. Some observational studies have shown that CABG is superior to PCI in terms of long-term outcomes [15, 16, 17], whereas another study indicated that the efficacy of PCI is comparable to that of CABG [18]. To date, none of the large-scale clinical trials, such as REVASC [19], EXPLORE [20], EURO-CTO [21], IMPACTOR-CTO [22], DECISION-CTO [23], and COMET-CTO [24], have demonstrated a benefit of PCI in major adverse cardiac and cerebrovascular events (MACCE) compared with CABG.

This study analyzed real-world data to compare the short- and long-term outcomes of CABG and PCI (with second-generation drug-eluting stents) in patients with CTO.

## 2. Methods

### 2.1 Study Population

**Fig. 1.**
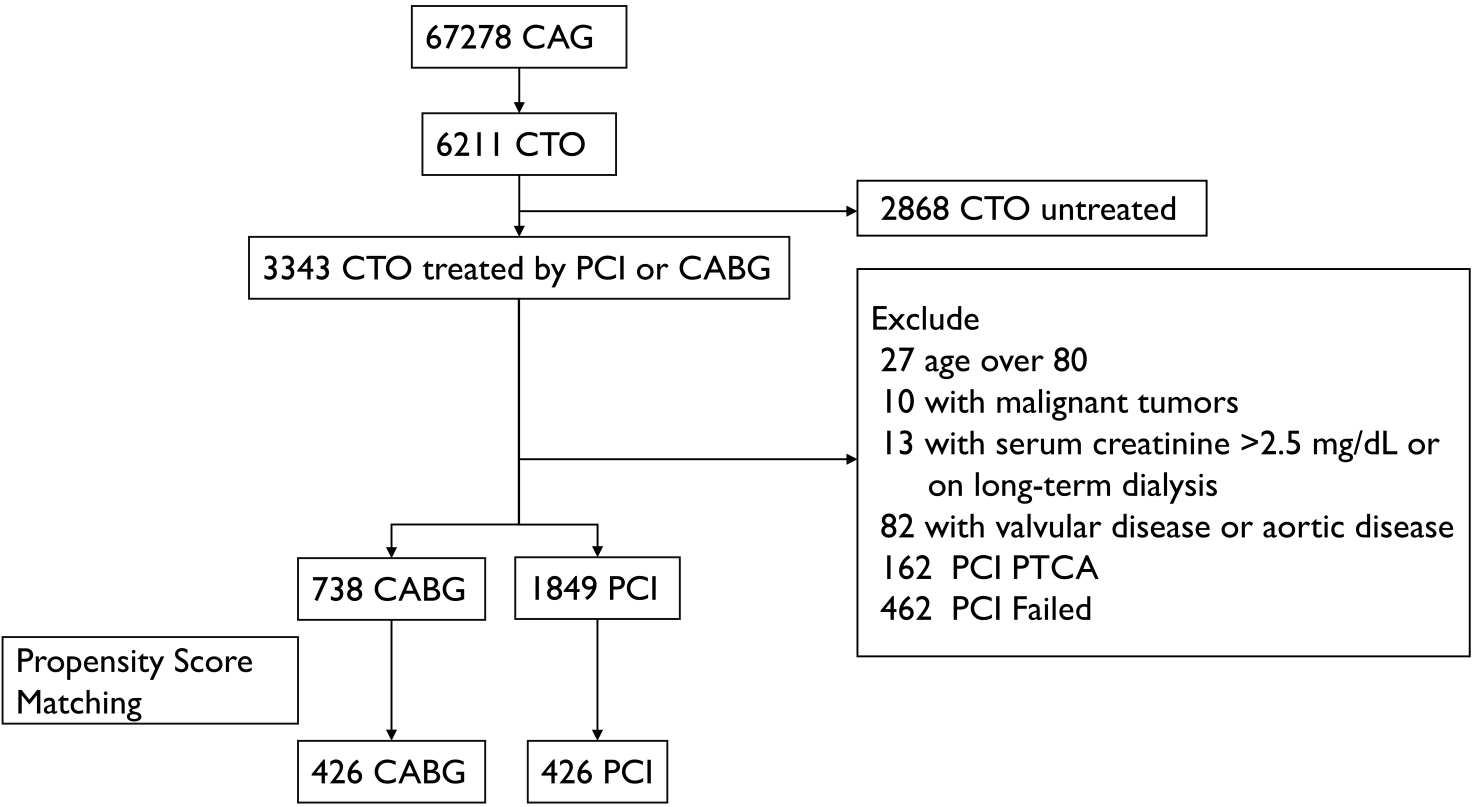
**Patient selection process and study protocol**. Abbreviations: CAG, coronary angiogram; CTO, chronic total occlusion; PCI, percutaneous coronary intervention; PTCA, percutaneous transluminal coronary angioplasty; CABG, coronary artery bypass grafting.

This retrospective study investigated patients with CAD who underwent coronary angiography at Beijing Anzhen Hospital (Beijing, China) from January 1, 2019 to December 31, 2021. Patients were diagnosed with definite CTO according to the Coronary Total Occlusion Academic Research Consortium (CTO-ARC) criteria [25] and underwent PCI (using second-generation drug-eluting stents) or CABG. Inclusion criteria were: (1) age between 18 and 80 years; (2) definite CTO with Thrombolysis in Myocardial Infarction (TIMI) 0 flow, no thrombus, no proximal contrast staining, established collateral circulation, and evidence of occlusion for more than 3 months; and (3) distal CTO vessel diameter ≥2.5 mm. Exclusion criteria were: (1) poor compliance, unable to adhere to antiplatelet therapy; (2) non-coronary or structural heart disease interventions within 30 days before or planned within 30 days after surgery; (3) previous CABG, valve surgery, or other major vascular surgeries; (4) renal failure with serum creatinine >2.5 mg/dL or on long-term dialysis; and (5) malignant tumors or life expectancy less than 1 year. The study protocol was approved by the Ethics Committee of Beijing Anzhen Hospital. The study flow is shown in Fig. 1.

### 2.2 Data Collection and Definitions

Baseline demographic, medical history, laboratory results, coronary anatomy, and surgical details were collected from the medical records. Follow-up data were collected from inpatient and outpatient records as well as telephone interviews. Baseline demographic and clinical characteristics, LVEF, and angiographic parameters were investigated from hospital records. Baseline creatinine levels were measured within 30 days before surgery. The estimated glomerular filtration rate was calculated using the Modification of Diet in Renal Disease formula. The diagnosis of chronic renal insufficiency is based on an estimated glomerular filtration rate <60 mL/min/1.73 m^2^. Echocardiography was performed within 30 days before PCI or CABG to measure the preoperative LVEF. Left main disease was defined as ≥50% stenosis of the left main coronary artery observed on angiography. Multivessel disease was defined as ≥70% stenosis in at least two of the three major epicardial coronary arteries. Complete revascularization was defined as achieving successful intervention (residual stenosis <30%) in all significant lesions (≥70% stenosis) in the three major coronary arteries and their primary branches. These assessments were made by the operating cardiologist through visual estimation. For CABG, complete revascularization was defined as providing bypass grafts to all major coronary arteries with ≥70% stenosis. All CABG procedures had no restrictions on the use of venous grafts.

### 2.3 Study Endpoints

The primary endpoints of this study included short-term (within 30 days post-operation) and long-term MACCE, which is a composite measure of all-cause mortality, cerebrovascular events (including ischemic stroke and hemorrhagic stroke), and myocardial infarction. Heart failure hospitalization and unplanned revascularization were considered secondary endpoints separately. The former was defined as rehospitalization with a primary diagnosis of heart failure after the initial surgery. The latter included any unplanned repeat PCI or CABG. Scheduled revascularizations within 90 days post-operation were not considered unplanned.

### 2.4 Statistical Analyses

**Fig. 2.**
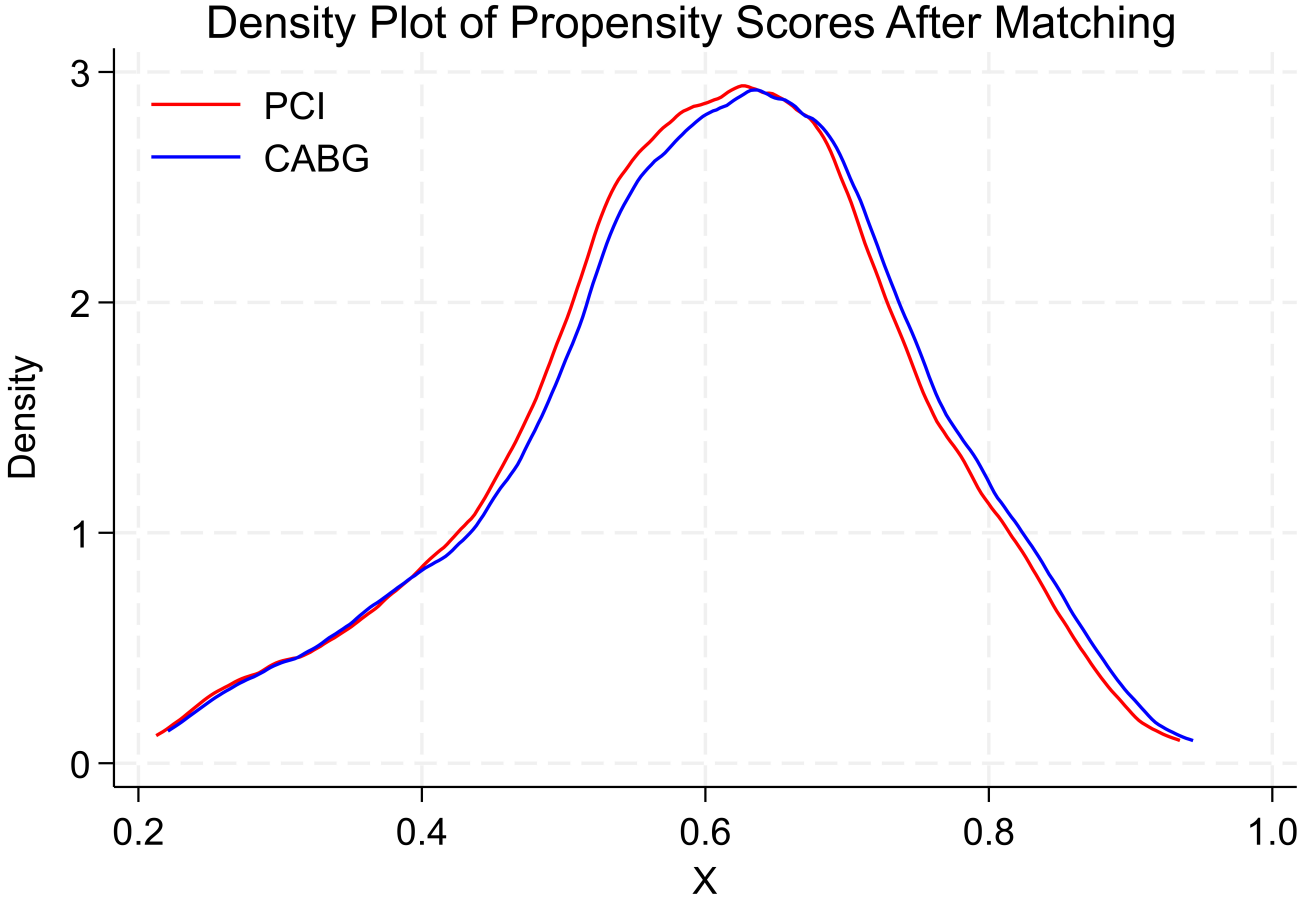
**Density plot of propensity scores after matching**.

Statistical analyses were conducted using Stata 18.0 (StataCorp LLC, College Station, TX, USA). Baseline characteristics between the PCI and CABG groups were balanced through 1:1 propensity score matching. This matching was performed using a nearest-neighbor algorithm with a caliper width of 0.1 times the standard deviation of the logit of the propensity score. Covariate balance between the groups was evaluated by calculating standardized mean differences. A standardized difference of less than 10.0% indicated an adequate balance between the two cohorts. To assess the overlap of propensity scores between the treatment (PCI) and control (CABG) groups after matching, kernel density estimation was used to plot the distributions. The resulting density plot visually demonstrated an overlap of propensity scores, confirming that balance was achieved between the groups (Fig. 2).

Given the low number of short-term events, Fisher’s exact test was applied to compare incidence rates between the two groups. Long-term clinical outcomes were evaluated using the Kaplan-Meier method to estimate cumulative incidence rates. Cox proportional hazards regression analysis was performed to assess the risk of outcomes of interest, with hazard ratios and 95% confidence intervals reported. Since all covariates achieved an SMD <10.0%, only the treatment strategy (PCI vs. CABG) was included in the Cox proportional hazards model. In the analysis of heart failure hospitalization and unplanned revascularization, all-cause death was treated as a competing event. Fine and Gray’s sub-distribution hazard regression model was used to estimate sub-distribution hazard ratios (SHRs) based on the cumulative incidence function, accounting for the impact of all-cause death on these outcomes. All statistical analyses were two-tailed, with a significance level set at *p* < 0.05.

## 3. Results

### 3.1 Baseline Characteristics

The study included 2587 patients with CAD with CTO who met the inclusion and exclusion criteria. Of these, 1849 patients (71.5%) chose PCI and received at least one second-generation drug-eluting stent, whereas 738 patients (28.5%) chose CABG. Before matching, differences were observed in baseline characteristics between the two groups. PCI patients were younger (58.29 ± 10.49 vs. 61.71 ± 9.11; *p* < 0.001), had higher body mass index (26.42 ± 3.36 vs. 25.95 ± 3.25; *p* = 0.002), higher ejection fraction (59.78 ± 8.59 vs. 58.02 ± 9.20; *p* < 0.001), and a lower proportion of cerebrovascular disease (11.25% vs. 18.56%; *p* < 0.001) and chronic kidney insufficiency (5.82% vs. 8.08%; *p* = 0.04). After 1:1 propensity score matching, 852 patients were included in the analysis, with 426 patients in each group. Among the 426 patients in the PCI group, bilateral coronary angiography was used in 201 procedures (47.2%). The antegrade wire escalation technique was applied in 325 procedures (76.3%), the antegrade dissection and re-entry technique was applied in 31 procedures (7.3%), the retrograde wire escalation technique was applied in 42 procedures (9.9%), and the retrograde dissection and re-entry technique was applied in 28 procedures (6.6%). Of the 738 patients who underwent CABG, one patient chose the right internal mammary artery for revascularization of the left anterior descending (LAD) artery, whereas two patients chose a saphenous vein graft. In the matched cohort, all CABG patients utilized left internal mammary artery (LIMA)-LAD grafts, achieving complete revascularization, whereas only 51% of patients (N = 217) achieved complete revascularization in the PCI group. Baseline characteristics before and after propensity score matching are illustrated in Table 1.

**Table 1.** **Baseline characteristics before and after propensity score matching**.

Patient Characteristics	Pre-matching				Post-matching			
	PCI	CABG	*p*	Standardized Difference (%)	PCI	CABG	*p*	Standardized Difference (%)
	(N = 1849)	(N = 738)			(N = 426)	(N = 426)		
Age, years	58.29 ± 10.49	61.71 ± 9.11	<0.001	34.80	61.45 ± 9.76	61.27 ± 8.98	0.78	1.95
Male	1505 (81.40)	594 (80.49)	0.59	2.31	338 (79.34)	342 (80.28)	0.73	2.34
BMI, kg/m^2^	26.42 ± 3.36	25.95 ± 3.25	0.002	14.12	26.04 ± 3.12	26.12 ± 3.29	0.74	2.32
Smoking	636 (34.40)	224 (30.35)	0.05	8.65	134 (31.46)	137 (32.16)	0.83	1.51
Alcohol Consumption	560 (30.29)	214 (29.00)	0.52	2.82	117 (27.46)	126 (29.58)	0.50	4.68
Hypertension	1304 (70.52)	531 (71.95)	0.47	3.15	306 (71.83)	314 (73.71)	0.54	4.22
Diabetes	817 (44.19)	316 (42.82)	0.53	2.76	183 (42.96)	190 (44.60)	0.63	3.31
Chronic Kidney Insufficiency	97 (5.25)	59 (7.99)	0.01	8.90	30 (7.04)	31 (7.28)	0.89	0.91
History of Myocardial Infarction	467 (25.26)	178 (24.12)	0.55	2.64	93 (21.83)	98 (23.00)	0.68	2.81
Cerebral Vascular Disease	208 (11.25)	137 (18.56)	<0.001	20.64	69 (16.20)	67 (15.73)	0.85	1.28
History of PCI	731 (39.53)	183 (24.8)	<0.001	31.94	113 (26.53)	119 (27.93)	0.64	3.16
LVEF, %	59.78 ± 8.59	58.02 ± 9.20	<0.001	19.22	58.63 ± 9.33	58.68 ± 9.07	0.93	0.56
LVSD, mm	32.97 ± 6.32	33.22 ± 6.68	0.22	3.74	33.12 ± 6.56	33.18 ± 6.57	0.89	0.97
Multiple CTO	221 (11.95)	185 (25.07)	<0.001	34.24	83 (19.48)	76 (17.84)	0.54	4.21
LAD CTO	813 (43.97)	284 (38.48)	0.01	11.16	172 (40.38)	167 (39.20)	0.73	2.40
Aspirin	1758 (95.08)	698 (94.58)	0.60	2.25	404 (94.84)	396 (92.96)	0.25	7.84
ADP Inhibitor	1712 (92.59)	685 (92.82)	0.84	0.88	388 (91.08)	389 (91.31)	0.90	0.83
Statin	1709 (92.43)	647 (87.67)	<0.001	15.94	376 (88.26)	374 (87.79)	0.83	1.45

Values are mean (SD) or No. of patients (%). Abbreviations: BMI, body mass index; LVSD, left ventricular end-systolic; LVEF, left ventricular ejection fraction; LAD, left anterior descending.

### 3.2 Short-Term Outcomes

**Fig. 3.**
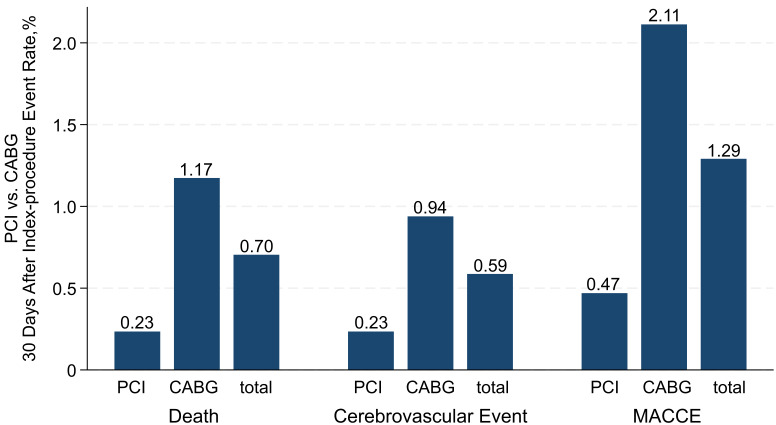
**Thirty days after the index procedure event rate**. Abbreviations: MACCE, major adverse cardiac and cerebrovascular events, assessed as all-cause death, cerebrovascular events, and myocardial infarction.

PCI patients had lower short-term MACCE rates compared to CABG patients. Within 30 days of the index procedure, there was one death (0.23%) in the PCI group and five deaths (1.17%) in the CABG group (*p* = 0.22); and one (0.23%) cerebrovascular event in the PCI group compared to four (0.94%) in the CABG group (*p* = 0.37). Overall, two (0.47%) MACCE were recorded in the PCI group, and nine (2.11%) in the CABG group (*p* = 0.06). No repeat revascularization, myocardial infarction, or heart failure hospitalization occurred in either group within 30 days of the index operation (Fig. 3).

### 3.3 Long-Term Outcomes

**Fig. 4.**
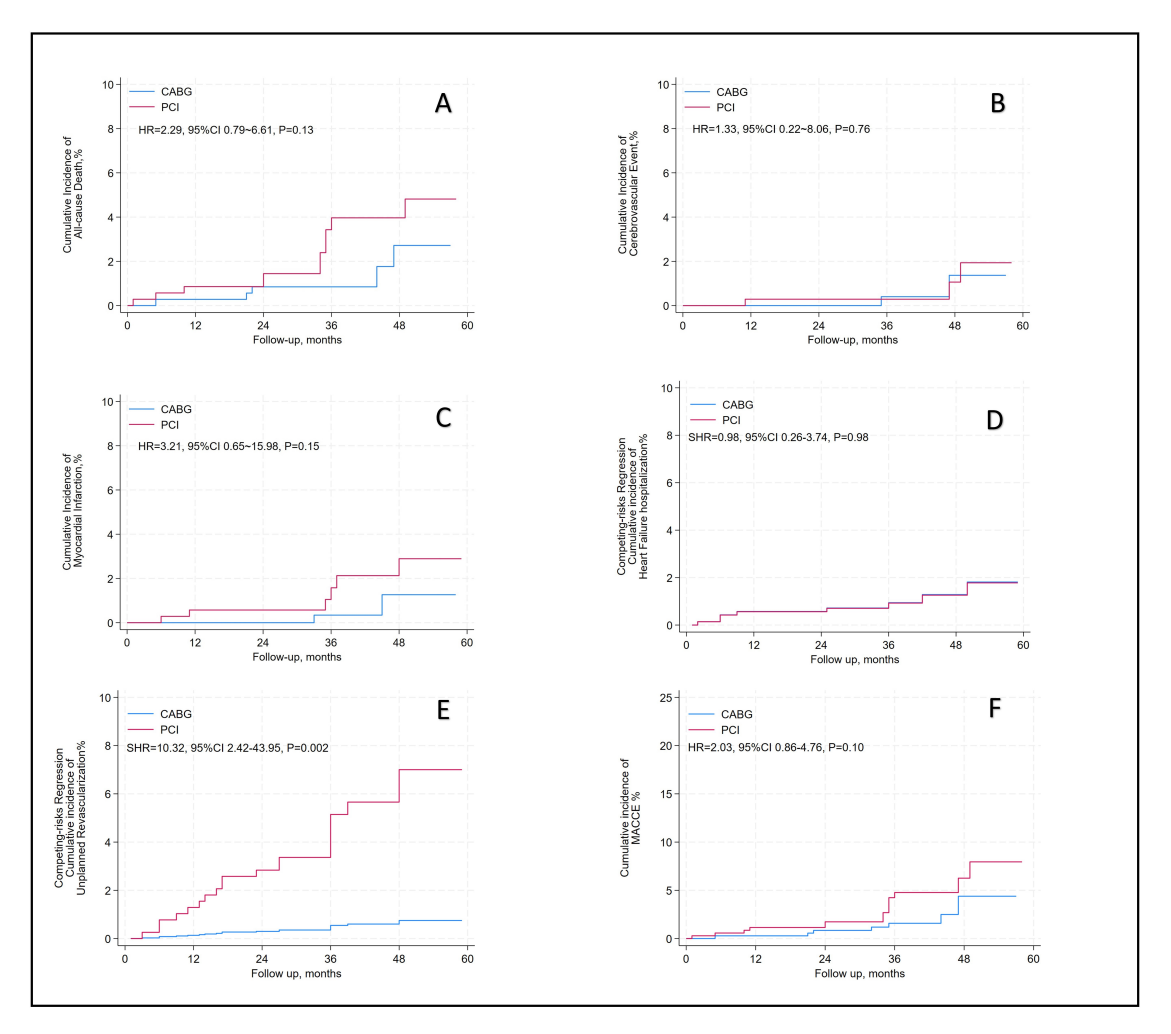
**Long-term clinical outcomes of PCI versus CABG**. (A) All-Cause Death. (B) Cerebrovascular Events. (C) Myocardial Infraction. (D) Heart Failure Hospitalization. (E) Unplanned Revascularization. (F) MACCE. Abbreviations: PCI, percutaneous coronary intervention; CABG, coronary artery bypass grafting; MACCE, major adverse cardiac and cerebrovascular events, assessed as all-cause death, cerebrovascular event, and myocardial infraction. The calculated sub distribution hazard ratio (SHR) for “heart failure hospitalization” and “unplanned revascularization” represents the SHR, with all-cause death considered the competing event.

After an average follow-up of 37.2-month, there were no differences between PCI and CABG in all-cause mortality (hazard ratio [HR] = 2.29, 95% confidence interval [CI]: 0.79–6.61; *p* = 0.13), cerebrovascular events (HR = 1.33, 95% CI: 0.22–8.06; *p* = 0.76), myocardial infarction (HR = 3.21, 95% CI: 0.65–15.98; *p* = 0.15). The incidence of MACCE was not significantly different (HR: 2.03, 95% CI: 0.86–4.76; *p* = 0.10). Hospitalization for heart failure, assessed with a subdistribution HR (SHR) to account for all-cause death as a competing event, also demonstrated no significant difference (SHR: 0.98, 95% CI: 0.26–3.74; *p* = 0.98). By contrast, unplanned revascularization was markedly higher in the PCI group, with an SHR of 10.32 (95% CI: 2.42–43.95; *p* = 0.002), indicating a statistically significant increase (Table 2 and Fig. 4).

**Table 2.** **Long-term clinical outcomes of PCI versus CABG**.

	HR	95% CI	*p* value
MACCE	2.03	0.86–4.76	0.10
All-cause death	2.29	0.79–6.61	0.13
Cerebrovascular event	1.33	0.22–8.06	0.76
Myocardial infarction	3.21	0.65–15.98	0.15
Heart failure hospitalization*	0.98*	0.26–3.74	0.98
Unplanned revascularization*	10.32*	2.42–43.95	0.002

CABG was set as reference to PCI. Abbreviations: CI, confidence interval; PCI, percutaneous coronary intervention; CABG, coronary artery bypass grafting; MACCE, major adverse cardiac and cerebrovascular events, assessed as all-cause death, cerebrovascular event, and myocardial infraction. * The calculated hazard ratio (HR) for “heart failure hospitalization” and “unplanned revascularization” represents the subdistribution HR, with all-cause death considered the competing event.

### 3.4 Subgroup Analyses

**Fig. 5.**
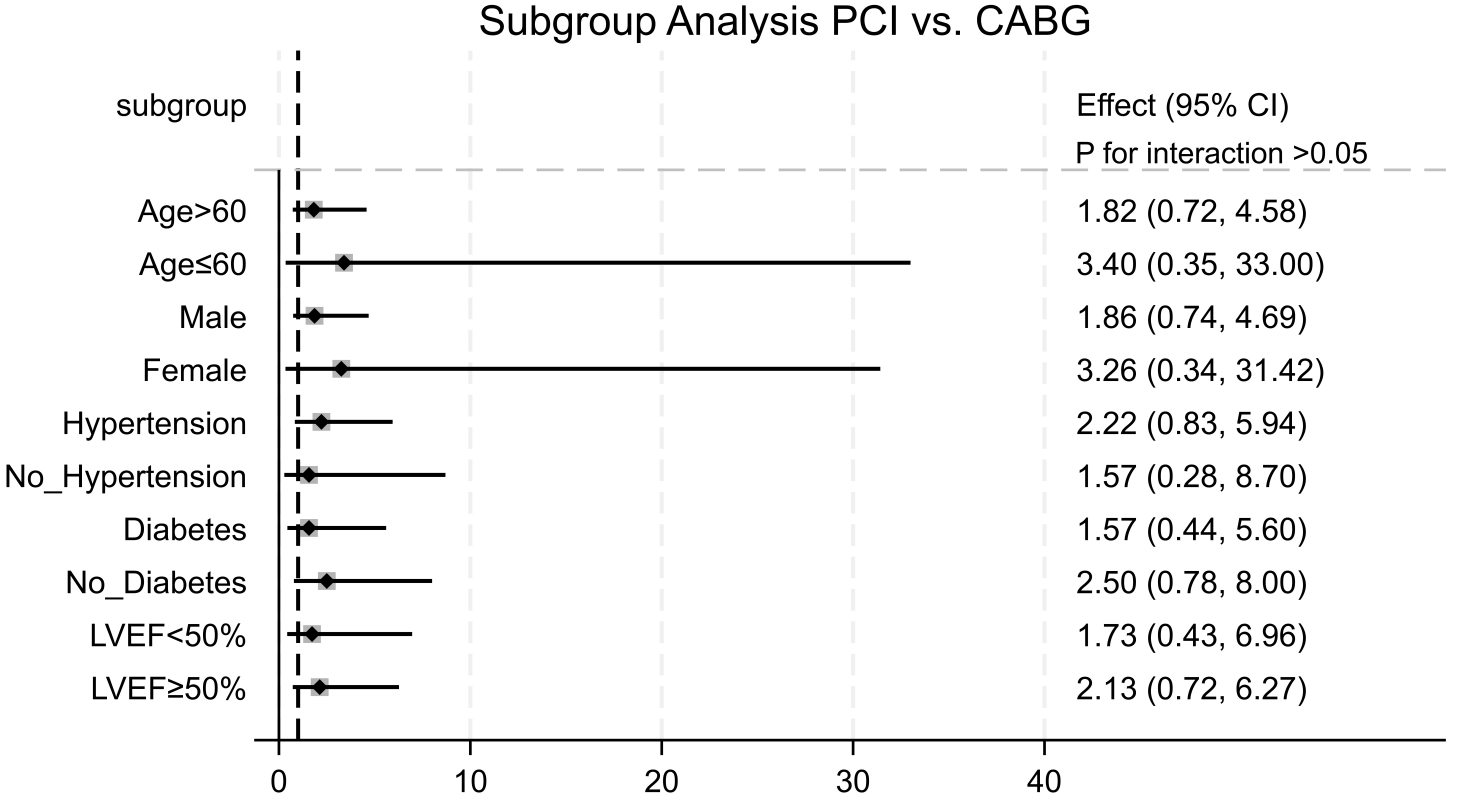
**Comparative hazard ratios of MACCE for subgroups of the PCI and CABG groups**. CABG was set as the reference group.

We conducted subgroup analyses to evaluate the potential association between treatment strategy and MACCE in different subpopulations. The comparative effectiveness of PCI and CABG showed no significant variation across subgroups, irrespective of age, sex, comorbidities (e.g., diabetes mellitus and hypertension), LVEF, or LAD-CTO (all *p* > 0.05) (Fig. 5).

## 4. Discussion

This large, single-center observational study analyzed real-world data to compare the short- and long-term clinical outcomes of different revascularization strategies in patients with CTO. Our study is one of the few to compare contemporary CTO PCI techniques with CABG in terms of safety and efficacy.

This study found that patients with PCI showed a trend toward a lower short-term MACCE rate compared those with CABG within 30 days of successful revascularization, consistent with the findings of a previous study [26]. The CABG group exhibited higher mortality in the early postoperative period, indicating a greater periprocedural risk.

Lin *et al*. [15] conducted a retrospective analysis of patients with CTO and multivessel disease, and found that PCI was associated with a lower 30-day mortality compared to CABG. Despite a higher baseline prevalence of cerebrovascular disease in the PCI group, the short-term cerebrovascular event rate remained significantly lower in this group (0.1% vs. 0.8%; *p *= 0.006). Our findings are in accordance with these results, further highlighting the advantage of PCI in reducing short-term adverse events.

Regarding long-term outcomes, PCI patients had a higher risk of repeat revascularization. Jang *et al*. [18] conducted an observational cohort study comparing second-generation drug-eluting stents with CABG. They found a higher risk of repeat revascularization in the PCI group at a median follow-up of 32 months compared to the CABG group, with consistent results after inverse probability weighting. Our study included a larger patient population, enhancing the robustness and generalizability of our findings. Notably, within our cohort, complete revascularization was achieved in 51.0% of PCI patients compared to 100% of CABG patients, which likely explains the higher rate of repeat revascularization observed in the PCI group.

Our study also revealed no significant differences between PCI and CABG in long-term MACCE. In Roth’s cohort, the survival curves for successful CTO-PCI and CABG became more parallel over time, suggesting comparable long-term outcomes between PCI and CABG [26]. In addition, Lin *et al*. [15] reported a higher risk of 5-year all-cause mortality, myocardial infarction, and cerebrovascular events in patients with CTO with multivessel disease treated with PCI compared to those treated with CABG. In their subgroup analysis, patients with PCI with three-vessel disease who achieved complete revascularization demonstrated comparable outcomes to those who underwent CABG, as measured by the composite endpoint of death, myocardial infarction, and cerebrovascular events.

The Synergy between PCI with Taxus and Cardiac Surgery Extended Survival (SYNTAXES) [27] extended follow-up study, one of the longest follow-up studies to date, reported no significant difference in long-term mortality between PCI and CABG over an average follow-up of 10 years. However, this study has been subject to several controversies. First, it was a post hoc analysis, which inherently carries methodological limitations. Second, the definition of occlusion used in the study deviates from currently accepted definitions, potentially impacting comparability. Furthermore, the PCI group predominantly utilized paclitaxel-eluting stents, representing a technological gap compared to contemporary drug-eluting stents. Finally, the PCI revascularization success rate was only 43.5%, significantly lower than the 60.5% achieved with CABG, underscoring the study’s limitations in drawing definitive conclusions.

Our study demonstrates that PCI offers advantages in short-term adverse events for patients with CTO but poses a higher long-term risk of unplanned revascularization. No significant differences were found in long-term MACCE between PCI and CABG.

## 5. Limitations

The study had several limitations. First, as a single-center, non-randomized observational study, it could not entirely eliminate the influence of confounding factors. Second, the study did not include detailed anatomical parameters or comprehensive operative data, nor did it account for medication adjustments during follow-up. Third, the follow-up did not involve functional tests, cardiac magnetic resonance imaging to assess viable myocardium or ischemic areas, or coronary CTA follow-up to evaluate long-term graft patency in patients with CABG.

## 6. Conclusion

In patients with CAD and CTO, PCI was associated with a trend of a lower short-term MACCE compared to CABG, but with a higher risk of unplanned revascularization. No significant differences were observed between PCI and CABG in terms of long-term all-cause mortality, MACCE, or heart failure hospitalization rates.

## Availability of Data and Materials

All data generated or analyzed during this study are included in this published article.

## References

[1] Masoomi R, Azzalini L (2023). Survival Following Recanalization of Chronic Total Occlusion: The Devil Is in the Details. *Journal of the American Heart Association*.

[2] Lawton JS, Tamis-Holland JE, Bangalore S, Bates ER, Beckie TM, Bischoff JM (2022). 2021 ACC/AHA/SCAI Guideline for Coronary Artery Revascularization: Executive Summary: A Report of the American College of Cardiology/American Heart Association Joint Committee on Clinical Practice Guidelines. *Circulation*.

[3] Abuzeid W, Zivkovic N, Elbaz-Greener G, Yaranton B, Patel V, Strauss B (2021). Association Between Revascularization and Quality of Life in Patients With Coronary Chronic Total Occlusions: A Systematic Review. *Cardiovascular Revascularization Medicine: Including Molecular Interventions*.

[4] Kucukseymen S, Iannaccone M, Grantham JA, Sapontis J, Juricic S, Ciardetti N (2023). Association of Successful Percutaneous Revascularization of Chronic Total Occlusions With Quality of Life: A Systematic Review and Meta-Analysis. *JAMA Network Open*.

[5] Sengodan P, Davies RE, Matsuno S, Chan AK, Kearney K, Salisbury A (2023). Chronic Total Occlusion Interventions in Patients with Reduced Ejection Fraction. *Current Cardiology Reports*.

[6] Perera D, Clayton T, O’Kane PD, Greenwood JP, Weerackody R, Ryan M (2022). Percutaneous Revascularization for Ischemic Left Ventricular Dysfunction. *The New England Journal of Medicine*.

[7] Assaf A, Diletti R, Hoogendijk MG, van der Graaf M, Zijlstra F, Szili-Torok T (2020). Vulnerability for ventricular arrhythmias in patients with chronic coronary total occlusion. *Expert Review of Cardiovascular Therapy*.

[8] Di Marco A, Anguera I, Teruel L, Dallaglio P, González-Costello J, León V (2017). Chronic total occlusion of an infarct-related artery: a new predictor of ventricular arrhythmias in primary prevention implantable cardioverter defibrillator patients. *Europace: European Pacing, Arrhythmias, and Cardiac Electrophysiology: Journal of the Working Groups on Cardiac Pacing, Arrhythmias, and Cardiac Cellular Electrophysiology of the European Society of Cardiology*.

[9] Cilia L, Megaly M, Davies R, Tehrani BN, Batchelor WB, Truesdell AG (2024). A non-interventional cardiologist’s guide to coronary chronic total occlusions. *Frontiers in Cardiovascular Medicine*.

[10] Watanabe H, Morimoto T, Shiomi H, Furukawa Y, Nakagawa Y, Ando K (2017). Chronic total occlusion in a non-infarct-related artery is closely associated with increased five-year mortality in patients with ST-segment elevation acute myocardial infarction undergoing primary percutaneous coronary intervention (from the CREDO-Kyoto AMI registry). *EuroIntervention: Journal of EuroPCR in Collaboration with the Working Group on Interventional Cardiology of the European Society of Cardiology*.

[11] Watanabe H, Morimoto T, Shiomi H, Kawaji T, Furukawa Y, Nakagawa Y (2018). Chronic total occlusion in non-infarct-related artery is associated with increased short-and long-term mortality in patients with ST-segment elevation acute myocardial infarction complicated by cardiogenic shock (from the CREDO-Kyoto AMI registry). *Catheterization and Cardiovascular Interventions: Official Journal of the Society for Cardiac Angiography & Interventions*.

[12] Sahu AK, Kazmi DH, Kaushik A (2024). Is it Worthy Enough to Revascularize Chronically Occluded Coronaries?. *Cardiology in Review*.

[13] Wang C, Liu S, Kamronbek R, Ni S, Cheng Y, Yan H (2023). Percutaneous Coronary Intervention versus Coronary Artery Bypass Grafting for Chronic Total Occlusion of Coronary Arteries: A Systematic Review and Meta-Analysis. *Journal of Interventional Cardiology*.

[14] Gong X, Zhou L, Ding X, Chen H, Li H (2021). The impact of successful chronic total occlusion percutaneous coronary intervention on long-term clinical outcomes in real world. *BMC Cardiovascular Disorders*.

[15] Lin S, Guan C, Wu F, Xie L, Zou T, Shi Y (2022). Coronary Artery Bypass Grafting and Percutaneous Coronary Intervention in Patients With Chronic Total Occlusion and Multivessel Disease. *Circulation. Cardiovascular Interventions*.

[16] Sun LY, Gaudino M, Chen RJ, Bader Eddeen A, Ruel M (2020). Long-term Outcomes in Patients With Severely Reduced Left Ventricular Ejection Fraction Undergoing Percutaneous Coronary Intervention vs Coronary Artery Bypass Grafting. *JAMA Cardiology*.

[17] Strauss BH, Knudtson ML, Cheema AN, Galbraith PD, Elbaz-Greener G, Abuzeid W (2021). Canadian Multicenter Chronic Total Occlusion Registry: Ten-Year Follow-Up Results of Chronic Total Occlusion Revascularization. *Circulation. Cardiovascular Interventions*.

[18] Jang WJ, Yang JH, Song YB, Hahn JY, Chun WJ, Oh JH (2019). Second-generation drug-eluting stenting versus coronary artery bypass grafting for treatment of coronary chronic total occlusion. *Journal of Cardiology*.

[19] Mashayekhi K, Nührenberg TG, Toma A, Gick M, Ferenc M, Hochholzer W (2018). A Randomized Trial to Assess Regional Left Ventricular Function After Stent Implantation in Chronic Total Occlusion: The REVASC Trial. *JACC. Cardiovascular Interventions*.

[20] Henriques JPS, Hoebers LP, Råmunddal T, Laanmets P, Eriksen E, Bax M (2016). Percutaneous Intervention for Concurrent Chronic Total Occlusions in Patients With STEMI: The EXPLORE Trial. *Journal of the American College of Cardiology*.

[21] Werner GS, Martin-Yuste V, Hildick-Smith D, Boudou N, Sianos G, Gelev V (2018). A randomized multicentre trial to compare revascularization with optimal medical therapy for the treatment of chronic total coronary occlusions. *European Heart Journal*.

[22] Obedinskiy AA, Kretov EI, Boukhris M, Kurbatov VP, Osiev AG, Ibn Elhadj Z (2018). The IMPACTOR-CTO Trial. *JACC. Cardiovascular Interventions*.

[23] Lee SW, Lee PH, Ahn JM, Park DW, Yun SC, Han S (2019). Randomized Trial Evaluating Percutaneous Coronary Intervention for the Treatment of Chronic Total Occlusion. *Circulation*.

[24] Juricic SA, Stojkovic SM, Galassi AR, Stankovic GR, Orlic DN, Vukcevic VD (2023). Long-term follow-up of patients with chronic total coronary artery occlusion previously randomized to treatment with optimal drug therapy or percutaneous revascularization of chronic total occlusion (COMET-CTO). *Frontiers in Cardiovascular Medicine*.

[25] Ybarra LF, Rinfret S, Brilakis ES, Karmpaliotis D, Azzalini L, Grantham JA (2021). Definitions and Clinical Trial Design Principles for Coronary Artery Chronic Total Occlusion Therapies: CTO-ARC Consensus Recommendations. *Circulation*.

[26] Roth C, Goliasch G, Aschauer S, Gangl C, Ayoub M, Distelmaier K (2020). Impact of treatment strategies on long-term outcome of CTO patients. *European Journal of Internal Medicine*.

[27] Kawashima H, Takahashi K, Ono M, Hara H, Wang R, Gao C (2021). Mortality 10 Years After Percutaneous or Surgical Revascularization in Patients With Total Coronary Artery Occlusions. *Journal of the American College of Cardiology*.

